# The Performance Evaluation Mechanism Based on Information Construction for Large Stand-Alone Medical Equipment and Its Support for Decision-Making of Purchasing

**Published:** 2020-01

**Authors:** Qing LU

**Affiliations:** Xuzhou Central Hospital, Xuzhou 221009, P.R. China

**Keywords:** Information construction, Large medical equipment, Stand-alone equipment, Hospital

## Abstract

**Background:**

Continuously deeper reform of public hospitals has put forward the need to innovate the philosophy and system of large medical equipment operation and management, and the phenomenon featuring “more attention to purchasing and less attention to management” need to be turned around.

**Methods:**

This research took use of information management to set up ID fields (unique number) for target stand-alone equipment; integrated statistics functions of HIS, PACS, LIS, RIS and equipment management system to get the basic operation data; informationized the work flow and reform technology to set up a post evaluation indictor system for the performance of stand-alone equipment; compared the service condition of newly purchased equipment with the feasibility application of relative department from various dimensions; designed objective post-evaluation indicators from various angles to scientifically manage existing medical equipment and support decision-making of new application for purchasing medical equipment.

**Results:**

Application performance of stand-alone equipment and clinical departments were ranked in a standardized manner. Decision-making mode based on data case of stand-alone equipment was set up. Net present value was evaluated. However, re-purchasing the instrument did not continuously increase the contribution of each instrument. The laboratory can purchase new instruments again, while the imaging department is not recommended to purchase.

**Conclusion:**

The performance evaluation mechanism based on information construction for large stand-alone medical equipment and its support for decision-making of purchasing is of great significance to improve the service life of equipment, exert the maximum effect and reduce economic waste.

## Introduction

Large medical equipment usually refers to medical equipment worth over $24450 USD, such as CT, MRT, DSA, DR, CR, LA and type-b ultrasound, which consist an important part of hospital asset management. However, as the phenomenon featuring “more attention to purchasing and less attention to management” prevails now, low utility of some equipment has caused wasting of resources (1–3). China now is at a critical phase of reforming public hospitals, to survive and develop along with cancelation of additional medicine price and implement of grading diagnosis and treatment, the original management mode, which is extensive, can no longer fit current situation. Hospitals must develop their inner construction by management refinement, issue identification and improvement proposals to raise the utility rate of resources and allocate resources in a scientific manner (4–6).

The philosophy and system of hospital operation and management need to actively adapt to the market economy and transform from pure attention to service to equal attention to service and benefit, so that they can continuously fit the competition development among hospitals and refine management philosophy. Therefore, management method featuring information and network is inevitable (7–9). Hospital managers and competent departments at all levels have raised increasingly higher requirements for the cost and performance analysis work for medical equipments, especially for those large ones (5). Good understanding of the performance of various large equipment plays a very important role as positive instruction and promotion to the reasonable usage and purchasing decision of medical equipment, which can even be applied to the hospital operation and management (4).

This research aimed to set up an evaluation mechanism for the post-performance of large stand-alone medical equipment during their whole life-circle. Besides, by implementing scientific, standard and elaborate calculation and analysis for the operation rate of medical equipment, this research was intended to achieve support for the decision-making of purchasing new medical equipment.

## Research Methods

**Fig. 1:**
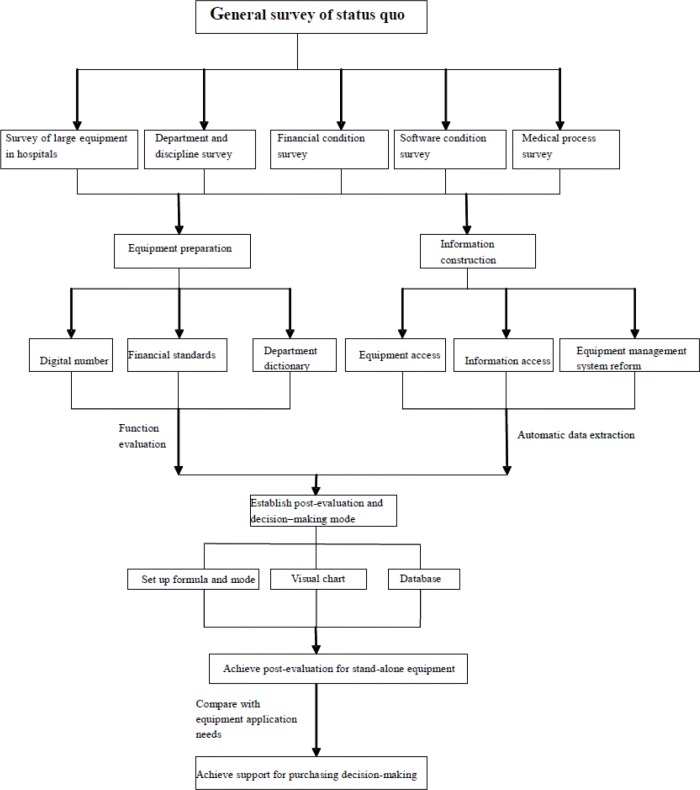
General survey of status quo

Large medical equipment worth over $28000 USD in our hospital were taken as research targets, which were assigned with ID fields (unique number); research tools include (10–12) information technology, net present value mode, decision-making tree mode, service ability plan, process analysis, Little’s law analysis, performance instrument panel and evaluation indicator (Fig. 1). We integrated statistics functions of HIS, PACS, LIS, RIS and equipment management system to get the basic operation data; informationized the work flow and reformed technology to set up a post evaluation indictor system for the performance of stand-alone equipment, and extracted data according to comprehensive, cost-effectiveness and validity principles (13, 14); compared the service condition of newly purchased equipment with the feasibility application of relative department from five dimensions including equipment performance, starting-up utility, development of equipment function, equipment maintenance and scientific and research articles (15–17); with discipline construction and hospital development goals in mind, designed objective post-evaluation indicators from various angles, including combination of quantitative and qualitative indicators, social and economic performance indicators (18, 19), so as to achieve scientific management for existing medical equipment and support decision-making of approving new application for purchasing medical equipment.

According to the above procedures, four departments of Orthopaedics, Interventional, Imaging (CT+ Magnetic Resonance) and Laboratory were selected from January 2015 to January 2017. For the large medical equipment newly purchased by each department (starting at the price of ≥ $71491 USD), the number of patients actually used, the total economic benefits generated, the maintenance costs due to the loss of the instrument, the final net income, and contribution of each instrument were counted. The abovementioned departments made a decision analysis on whether to purchase large medical equipment again from 2017 to 2019. There were two strategies, one was to continue to purchase large equipment, and the other was to decide whether to buy and how much to buy according to the benefit ratio of the previous year. Then the benefit ratio of the newly purchased large medical equipment from 2017 to 2019 was compared.

### Implement Details

1. Use device management software to assign ID field (unique number) to large stand-alone medical equipment.
2. Informationized work flow and reform technology to integrate HIS, OA, PACS, LIS, RIS, equipment management system and other software to remove information isolated island.
3. For stand-alone equipment whose access support data extraction, define dictionary for equipment department and import data source to the equipment management system.
4. For stand-alone equipment whose access do not support data extraction, define function according to service project, finance, HIS, OA, PACS, LIS and RIS to deduct and import data source to the equipment management system.
5. Design formula and analysis mode according to evaluation and decision indicators to generate visual chart.
6. Improve data collection and decision-making mode according to practical operation results.

This research took one year to be completed. In the first month, arrangement of equipment data was done and standing book and digital coding were set up; in the second month, we used management tool to define statistic parameters and set up relative models; in the third month, ultrasonic equipment were taken as the trial unit to finish the information construction, flow reform and synchronous data access; in the fourth month, we developed post evaluation model for stand-alone equipment, proceeded synchronous data import, completed data evaluation, quantized following indicators for stand-alone equipment as operation income, net income, benefit rate, service person-time, usage rate, out-of-service days, working days, recoverable value, and rate of return, so that finally generate monthly database view and chart; in the fifth month, we evaluated the function, fix issues and created workable project goals; from the sixth to the twelfth, we implemented post evaluation for large stand-alone equipment of other departments.

## Results

Flow re-creation and information construction enabled automatic and daily summary of data of stand-alone equipment and supported manual extraction of the latest data, so that the promptness and accuracy of stand-alone equipment data were secured.

Data mining was used to accurately analyze the data source of stand-alone equipment, quantize following indicators for stand-alone equipment as operation income, net income, benefit rate, service person-time, usage rate, out-of-service days, working days, recoverable value, and rate of return to finally generate monthly database view and chart.

Application performance of stand-alone equipment and clinical departments were ranked in a standardized manner.

Decision-making mode based on data case of stand-alone equipment was set up. Net present value was evaluated by comparing the project budget of equipment application, usage expectation and cost estimation, so as to support the decision-making of purchasing new medical equipment.

### Data analysis

Instruments purchased between January 2015 and January 2017 were selected.

Orthopedics Department: 2 arthroscopy instruments ($96997 and $106982), 2 discoscopy instruments ($156907 and $136937) and 2 digital CT imaging systems ($84159 and $89865).

Interventional Department: 3 digital X-ray imaging systems ($78453, $79880 and $81306).

Imaging Department: 5 CT inspection instruments ($81306, $85586, $88438, $92718 and $84159) and 3 Magnetic resonance instruments ($95570, $106982 and $111261)

Laboratory Department: 2 automatic analysis systems ($71321 and $74174).

The total income and total loss of each department were counted.

### Orthopedics Department

Overall, 450 new patients benefited from instruments newly purchased, with a total income of $2246630. The average cost per patient was $4978, the total loss was $38371; the net income was $2201840, and the total expenditure was $671849. The revenue was $1530492, and the average value of each instrument was $254903.

### Interventional Department

The newly purchased instruments benefited 562 patients, with a total income of $1002068. The average cost per patient was $1783, the total loss was $19256, and the net income was $982811. The total expenditure was $239640, and the final income was $743170. The average contribution value per instrument was $247728.

### Imaging Department

The new acquisition of instruments benefited a total of 1025 patients, with a total income of $862634. The average cost per patient was $841, the total loss was $306,000, and the net income was $818985. The total expenditure was $746023, the final income was $72961, and the average contribution value per instrument was $9114.

### Laboratory Department

The new instrument benefited 1326 patients, with a total income of $605263. The average cost per patient was $456, and the total loss was $26816. The net income was $578446, the total expenditure was $145496, and the final income was $432950. The average contribution value per instrument was $216475.

According to the calculation results, we believe that the orthopedics and interventional departments should purchase the new instrument again from March 2017 to March 2019. The laboratory can purchase the new instrument, while the imaging department should not purchase the new instrument again.

Orthopedics department purchased 1 arthroscopy instrument, 1 discoscopy instrument and 1 digital CT imaging system at the price of $99850, $139790 and $85585 respectively. A total of 156 patients were benefited, with a total income of $747678. The average cost of per patient was $4792, the total loss was $14977; the net income was $758376, the total expenditure was $325226; the final income was $407474, and the average contribution value per instrument was $135824.

Interventional department purchased a digital X-ray imaging system at a price of $79880, benefiting a total of 102 patients. The total income was $162955, the average cost per patient was $1597; the total loss was $12695, the net income was $150260; the total expenditure was $79880. The final income was $70380, and the average contribution value per instrument was $70380. It can be seen that the continuous purchase of large medical equipment cannot continue to increase the contribution value of the instrument in the short term.

The laboratory department decided to purchase a fully automated analysis system at a price of $72748, benefiting 525 patients, with a total income of $209685. The average cost of per patient was $39940, the total loss was $9842, and the net income was $199843. The total expenditure was $72748, and the final income was $127095. The average contribution value per instrument was $127095. Therefore, it is feasible for the laboratory to purchase the instrument again.

The imaging department purchased 2 CT instruments and 1 magnetic resonance instrument. The price of CT instruments was $81306 and $85585 respectively, and the price of the magnetic resonance instrument was $99850. A total of 465 patients were benefited, with a total income of $371442. The average cost of per patient was $798, the total loss was $31666; the net income was $340587, the total expenditure was $267379, and the final income was $73207. The average contribution value per instrument was $24450. It can be seen that the contribution value of each instrument is still low, which may increase the medical cost input and not increase the medical income relatively.

## Discussion

### Analysis of cost and performance status quo of stand-alone equipment

Most analysis was carried out from financial aspect, or oriented to theoretical exposition and research approach (20). The key points of the research lied in evaluation indicators of economics by comparing cost-effectiveness. However, there is just a few of evaluation system in our country having tried analysis of stand-alone equipment. Economic calculation is carried out based on insufficient database and it is not common to see literature about systematic research on the collection of objective cost-effectiveness data of stand-alone equipment and relative analysis methods. Along with introduction of various information systems to hospitals, we have gradually get ready with the conditions to collect objective cost-effectiveness data of stand-alone equipment (21). Research of Chia-Hung Chien et al from Taiwan has discussed how to use internet information system to manage the service/effectiveness conditions of medical equipment (22). Walid Tarawneh et al have reported application of information maintenance and management platform of the Heath Department to analyze and evaluate the downtime of medical equipment within the system (23). However, because of absence of continuous objective record of service condition of stand-alone equipment, various information systems are often developed by different information providers, which have caused low integrality and difficulty to sharing among different systems. There are still all kinds of block issues for data extraction (24, 25).

### Application effect of informationized management

This research has got following conclusions: flow re-creation and information construction enables automatic summary of data of stand-alone equipment every day and support manual extraction of the latest data, so that the promptness and accuracy of stand-alone equipment data are secured; stand-alone equipment data source can be accurately analyzed from various aspects, including operation income, net income, equipment benefit rate and other indicators to a generate view and form of monthly data base; Application performance of stand-alone equipment and clinical departments are ranked in a standardized manner; Decision-making mode based on data case of stand-alone equipment is set up. Net present value is evaluated by comparing the project budget of equipment application, usage expectation and cost estimation, so as to support the decision-making of purchasing new medical equipment. Establishment and implement of such mechanism will improve the phenomenon of insufficient feasibility analysis of the need for purchasing equipment during budget application phase and change the phenomenon featuring “more attention to purchasing and less attention to management”. Issues such as idle equipment after purchasing, low utility, limited function development and absence of target patients will be prevented and waste of budget for hospital equipment will be effectively controlled, so that hospital’s service ability and effectiveness to patients will be promoted to further improve the satisfaction rate of patients and strengthen the competitiveness of hospitals (26).

Establishment and implement of cost and performance analysis mechanism of stand-alone medical equipment in hospitals have positive meanings for the scientific management of medical equipment from following aspects (27): 1. Scientific performance analysis mechanism of stand-alone medical equipment plays a positive role in achieving economic, quantitative and digital management of medical equipment and overcome previous shallow management decided by impression and experience in a qualitative manner. 2. Such mechanism can help hospital departments to set up a whole picture of cost accounting, economic benefits and social benefits, overcome benefit-oriented thought or pursue of high level equipment without consideration to cost. 3. Such mechanism will reduce the vulnerability and waste, improve economic benefit, decrease operation cost, and it is definitely necessary for promoting the management level and competitiveness of hospitals. 4. This mechanism is also of important reference value for clinical path research and research on and reform of relative hospital policies.

### Problems found in performance management of stand-alone equipment

Methods to extract income data of stand-alone equipment and part of cost data still require improvement. Data of some examination equipment and information management system are closely related, therefore, relative usage data of these equipment require unified compatibility among parameters and standards.

Indicator model system of stand-alone equipment cost and performance requires improvement as relatively unified standards or regulations have not been established yet. Along with continuous implement of relative work, on the one hand, we need to gradually set up relatively standard basic analysis and evaluation methods and indicators. On the other hand, we need to positively explore differences among evaluation indicators among various equipment, such as examination and treatment type, non-consumable and consumable type, earning type and tool type equipment and so on. And analysis and evaluation methods for equipment which cannot generate direct cost need future exploration.

Unified thought is failed to be arrived. The hospital has not developed a unified standard during detailed operation process, failed to define specific regulations and implement steps about how to manage performance of stand-alone equipment. Most management work are still pending at the experience phase, starting analysis from the perceptual knowledge, lacking scientific evaluation methods.

Therefore, the establishment of scientific and effective cost and performance evaluation mechanism of stand-alone equipment need help from following aspects (28). Overall hospital conditions. Medical equipment plays an important role in the discipline construction and overall development of hospitals. It is a significant task to operate and manage medical equipment in a qualified way with high effectiveness and this needs strong support from hospital leaders.

Department requirements. Scientific equipment application and decision serve as a good insurance for the discipline construction and department development, which need positive cooperation among clinical departments.

Hardware conditions. Our hospital now owns about 300 large medical equipment worth at over 24450 USD. Equipment samples have great diversity, basically covering industry characteristics.

Research achievements have relatively strong reliability and practical value.

Software conditions. Original hospital equipment management software can be taken as the basis to develop post evaluation module and decision-making support module for stand-alone equipment.

In the aspect of project team, the team needs at least two MBA (medical major and software engineering major) and one financial person with knowledge of management and accounting theory and rich practical experience.

In the aspect of earlier stage preparation, this research has made detailed and feasible solutions named *Solutions to Evaluation and Management of Stand-alone Hospital Ultrasonic Equipment*, whose operation process, implement experience and results are expected to be leveraged by other large medical equipment.

### Research innovation points and expectations

It is difficult to evaluate stand-alone medical equipment during their whole life circle, especially to collect objective data, which has led to poor real-time post evaluation indicators. This research has innovatively applied information method to combine target stand-alone equipment with the establishment of post evaluation mechanism. Therefore, it achieves real-time collection of stand-alone equipment data and management of corresponding information module. Besides, the mechanism has regulated the feasibility of equipment application, unified evaluation standards, and standardized evaluation methods. Stand-alone equipment has been assigned with corresponding project name, charging standards and ID coding, which contributes to a scientific post evaluation mechanism for stand-alone equipment. Management goals, such as summarizing hospital medical equipment performance and statistics, have improved the inherent evaluation mode of medical equipment which is relatively extensive in the industry. And the mechanism has provided support to purchasing and decision-making of new medical equipment.

## Conclusion

Though it is pretty hard to achieve scientific, sound and effective cost and performance evaluation system for stand-alone equipment, departments of equipment, finance and information of hospitals at all levels are now trying their best to keep working out solutions. This research, by analysis of cost and performance of stand-alone equipment, can achieve scientific quantitative evaluation and analysis of performance and usage of medical equipment, especially those large ones, so that performance management work of various medical equipment can proceed on a normalized route with virtuous circle, and to the best give play to social and economic benefit.

We will go on promoting post evaluation and decision-making support to all areas and all stand-alone equipment among our hospital, and strive for the approval of municipal health and family planning commission, municipal science and technology commission and National Natural Science Foundation of China, and continue to strive for the standardization of industry standards of medical equipment management.

## Ethical considerations

Ethical issues (Including plagiarism, informed consent, misconduct, data fabrication and/or falsification, double publication and/or submission, redundancy, etc.) have been completely observed by the authors.
